# Sexual behaviour of women in rural South Africa: a descriptive study

**DOI:** 10.1186/s12889-016-3207-6

**Published:** 2016-07-12

**Authors:** Jan Henk Dubbink, Lisette van der Eem, James A. McIntyre, Nontembeko Mbambazela, Geoffrey A. Jobson, Sander Ouburg, Servaas A. Morre, Helen E. Struthers, Remco P. H. Peters

**Affiliations:** Anova Health Institute, Johannesburg, Tzaneen South Africa; Institute for Public Health Genomics (IPHG), Department of Genetics and Cell Biology, Research School GROW (School for Oncology & Developmental Biology), Faculty of Health, Medicine & Life Sciences, University of Maastricht, Maastricht, The Netherlands; Department of Medical Microbiology & Infection Control, Laboratory of Immunogenetics, VU University Medical Center, Amsterdam, The Netherlands; School of Public Health and Family Medicine, University of Cape Town, Cape Town, South Africa; Division of Infectious Diseases & HIV Medicine, Department of Internal Medicine, University of Cape Town, Cape Town, South Africa; Department of Medical Microbiology, University of Pretoria, Pretoria, South Africa; Department of Medical Microbiology, Maastricht University Medical Center, Maastricht, The Netherlands; Anova Health Institute, PostNet Suite 242, Private Bag X30500, 2041 Houghton, Johannesburg South Africa

**Keywords:** Sexual behaviour, Age, Ethnic group, HIV, Women, Rural, South Africa

## Abstract

**Background:**

Sexual behaviour is a core determinant of the HIV and sexually transmitted infection (STI) epidemics in women living in rural South Africa. Knowledge of sexual behaviour in these areas is limited, but constitutes essential information for a combination prevention approach of behavioural change and biomedical interventions.

**Methods:**

This descriptive study was conducted in rural Mopani District, South Africa, as part of a larger study on STI. Women of reproductive age (18–49 years) who reported sexual activity were included regardless of the reason for visiting the facility. Questionnaires were administered to 570 women. We report sexual behaviour by age group, ethnic group and self-reported HIV status.

**Results:**

Young women (<25 years) were more likely to visit bars, practice fellatio, have concurrent sexual partners and report a circumcised partner than older women (>34 years); there was no difference for condom use during last sex act (36 % overall). Sotho women were more likely to report concurrent sexual partners whereas Shangaan women reported more frequent intravaginal cleansing and vaginal scarring practice in our analysis. HIV-infected women were older, had a higher number of lifetime sexual partners, reported more frequent condom use during the last sex act and were more likely to have a known HIV-infected partner than women without HIV infection; hormonal contraceptive use, fellatio, and a circumcised partner were less often reported.

**Conclusions:**

This study provides insight into women’s sexual behaviour in a rural South African region. There are important differences in sexual behaviour by age group and ethnicity and HIV status; these should be taken into account when designing tailor-made prevention packages.

## Background

Sexual behaviour is the most important determinant of the burden of HIV and sexually transmitted infections (STI) in South Africa [1]. This country has generalized HIV and STI epidemics that rank among the highest in the world; HIV prevalence is estimated to be 17.8 % among women of reproductive age [2–4]. These epidemics are largely driven by heterosexual contact but key and vulnerable populations should not be ignored [3, 5].

In recent years many strategies have been employed to reduce the burden of HIV and STIs in Africa including the distribution of free male and female condoms, financial incentives for abstinence programmes, roll-out of antiretroviral therapy (ART) and promotion of voluntary medical male circumcision. Also, the potential impact of mass treatment of STIs, antiretroviral pre-exposure prophylaxis, and topical microbicide use has been explored. However, efforts to modify sexual behaviour, one of the core determinants of these epidemics, have been relatively limited despite the acknowledged importance of a combination approach of behavioural change and biomedical interventions [6–9]. Initiatives to modify sexual behaviour for prevention are important. For example, a review demonstrated that the majority of the countries with a reduction in HIV transmission were accompanied by changes in sexual behaviour [10]. A recent study from sub-Saharan Africa showed the importance of targeting the general sexually active population and not only HIV serodiscordant couples because of the large contribution of extra-couple transmission to new HIV infections [11].

Sexual behaviour of women varies across the globe and over time, including in Africa [4, 12]. Efforts to monitor the HIV epidemic are complicated by the geographical evolution of many sub-epidemics at provincial and district level, partly associated with differences in geographic characteristics [3, 13]. Important determinants of variation in sexual behaviour include setting (urban or rural), age, socio-economic level, ethnic group and HIV status [14]. A recent review of sexual behaviour of women in sub-Saharan Africa, excluding South Africa, showed that women with lowest education and those living in more rural environments were of younger age at sexual debut and had more pregnancies [14]. A study from Uganda showed significant differences in sexual behaviour of women living in rural compared to urban regions with rural women having more often a partner that is more than 10 years older, reporting less frequent condom use and to know their HIV status [15]. Studies from South Africa show differences in sexual behaviour (i.e. alcohol use, unprotected intercourse and concurrency) between ethnic groups [16, 17]. A recent study showed a higher point prevalence of concurrent sexual partnerships among the ethnic groups most affected by HIV in South Africa [18]. Finally, HIV-status may influence sexual behaviour towards higher or lower risk behaviour: a recent study showed a decline of unprotected sex after HIV-positive diagnosis and a further decline after awareness of HIV discordance, but other studies have shown an increase in risk behaviour associated with HIV-infection [10, 19–21]. Information about sexual behaviour is warranted to inform the design of prevention messages and programmes as one size may not fit all. Information about specific groups is required to build cultural competence in healthcare and therewith different opportunities and entry points for prevention messaging. For example, messages for adolescent discussion groups (age) could be different from those at the HIV clinic (HIV status) and could vary by geographic region (different ethnic groups). In this study we describe sexual behaviour reported by women living in rural South Africa in relation to age, ethnicity and HIV status. Furthermore, associations with condom use are explored.

## Methods

### Design

This study was embedded in a cross-sectional study conducted at 25 of the 100 primary healthcare (PHC) facilities across Mopani District, Limpopo Province, South Africa [22]. Data from all 25 facilities was included in this evaluation with the number of participants ranging from 8 to 52 per PHC facility. Mopani District is one of the most rural and poorest districts in South Africa with high rates of poverty and illiteracy; Shangaan and Sotho are the two largest ethic groups in this district [23]. In brief, the facilities were selected based on wide geographic location and size and diversity of catchment population. We visited each PHC facility on a random weekday from November 2011 to February 2012. Group information about the study was provided in the waiting area followed by individual counselling of women who were interested in participating, regardless of the reason for visiting the facility that day. Women aged 18–49 years who reported sexual activity during the previous six months were eligible. After obtaining written informed consent, clinical and sexual histories were taken by healthcare workers (through an English paper-based questionnaire translated into the language of the participant). These healthcare workers were carefully selected from the staff of Anova Health Institute, as they had to be all proficient in the English language and at least one local language and were specifically trained to obtain information about sexual characteristics of the participants. This analysis is limited to women of Sotho (*n* = 310) and Shangaan (*n* = 260) ethnicity (94 % of participants in the original study; 34 women were of other ethnicity including Venda, Zulu and Shona. The Human Ethics Research Committee of the University of the Witwatersrand, South Africa, approved the study (Ref M110726).

### Clinical criteria and definitions

Information collected in this study was self-reported including HIV status (although counselling and testing were offered). HIV-status was classified positive when the participant reported to be HIV-infected, negative in case of a negative HIV test less than six months ago, and unknown in other cases. Sexually active in the last 6 months was defined as a consensual or non-consensual vaginal, or anorectal intercourse, or fellatio in the 6 months prior to the inclusion day. Other data collected included demographic characteristics, general behaviour (e.g. alcohol use, intravaginal cleansing), risk-taking behaviour (e.g. condom use, transactional sex), sexual practices, sexual contacts, and specific questions about the steady sexual partner. Most indicators were self-explanatory; however the following definitions were used. Sexual relationships were considered concurrent if sexual partnerships overlapped in time. Sex with multiple partners was defined as sexual contact with more than one partner at the same time and place. Coercion was considered present when the participant reported social or psychological pressure to have sex; physical force used to have sex was defined as rape. Vaginal cleansing was defined as using a douche or another device to clean the vagina with water and possibly herbs and/or soap. Vaginal scarring practice was defined by report of vulvar scars that was confirmed on examination. These scars relate to a ritual that may be performed after birth or for treatment purposes by a traditional healer.

### Data analysis

Data were double-entered in Epi Data (Epi Info™ version 3.5.3) and analysed using SPSS version 20.0 (SPSS Inc., Chicago, USA). Determinants of sexual behaviour were compared between women of different age groups, ethnic group, HIV-status, and for condom use. Data are provided as numbers with proportion (%) and as median with range. Dichotomous data are compared between groups using Chi-squared test or Fisher’s Exact test if appropriate, whereas continuous data were compared between two groups using the Mann Whitney U test and between multiple groups using the Kruskal Wallis test. Univariate analysis was performed to examine possible differences in sexual behaviour between age groups (<25 years, 25–34 years, and >34 years), for ethnic origin (Sotho and Shangaan) and HIV-status (positive vs. negative), and to determine factors of condom use. A *p*-value of equal to or less than 0.05 was considered to be statistically significant.

## Results

### Characteristics of study population

Three hundred and ten women (54 %) were of Sotho and 260 (46 %) were of Shangaan ethnic origin (Table 1). The median age of participants was 30 years (range 18–49), which was similar for Sotho and Shangaan women. HIV infection was reported by 178 (31 %) participants and was similar for Sotho (29 %) and Shangaan (34 %) women (*p* = 0.62). Women in the oldest age group were significantly more likely to be HIV-infected (*p* < 0.001).

**Table 1:** Characteristics of study participants (*n* = 570)

Characteristic	Total (570)
Age	30 (18–49)
Ethnicity	
Sotho	310 (54)
Shangaan	260 (46)
HIV-status	
HIV-infected	178 (31)
HIV-uninfected^a^	257 (45)
Unknown	131 (23)
On ART if HIV-infected	
On ART	94 (53)
Not on ART	84 (47)
Marital status	
Single	301 (53)
Married/engaged	240 (42)
Divorced/widow	28 (4.9)
Unemployed	424 (75)
Pregnant	
Yes	98 (17)
No	472 (83)

Data are provided as numbers (%) and median (range). HIV, human immunodeficiency virus; STI, sexually transmitted infection

^a^less than six months tested negative

### Age and sexual behaviour

Younger women reported more frequently to visit bars, practice fellatio (but not receptive anal intercourse (RAI)), report concurrent partners and report a circumcised partner than older women (Table 2). Older women were more likely to have a partner more than 10 years older, know that their partner had concurrent partners and know his HIV status. The latter association was only manifested for HIV-infected women (*p* < 0.001) whereas there was no association between age and knowledge of the partner’s HIV status for HIV-negative women.

**Table 2:** Characteristics of sexual behaviour of women by different age-groups (*n* = 569)

Characteristic	<25 years (*n* = 155)	25-34 years (*n* = 210)	>34 years (*n* = 204)	*p*-value
General characteristics				
HIV infected	20 (17)	74 (47)	84 (52)	<0.001
Hormonal contraceptives	63 (41)	86 (41)	75 (37)	0.66
Intravaginal cleansing	34 (22)	47 (23)	54 (27)	0.50
Vaginal scarring practice	6 (3.9)	15 (7.1)	14 (6.9)	0.38
Visits bars*	33 (22)	26 (12)	17 (8.4)	0.001
Alcohol use [before sexual intercourse]	18 (12)	26 (12)	12 (5.9)	0.061
Sexual practice in past 6 months				
Currently has a steady partner	146 (95)	199 (96)	192 (95)	0.92
Currently has occasional partner(s)	16 (10)	26 (13)	21 (10)	0.73
Condom use during last sex act	57 (37)	73 (35)	76 (37)	0.86
Receptive anal intercourse	9 (6.0)	10 (5.0)	7 (3.7)	0.60
Practices fellatio^	28 (19)	34 (17)	14 (7.3)	0.003
*HIV +*	*3 (16)*	*8 (11)*	*5 (6.6)*	*0.18*
*HIV -*	*21 (23)*	*19 (24)*	*6 (8)*	*0.021*
Sexual contact in past 12 months				
Concurrent sexual partners^a^	25 (16)	45 (22)	17 (8.4)	0.001
*HIV +*	*2 (11)*	*17 (24)*	*23 (28)*	*0.18*
*HIV -*	*14 (15)*	*16 (19)*	*3 (3.8)*	*0.048*
Sexual partner > 10 years older^b^	27 (18)	40 (19)	59 (29)	0.014
*HIV +*	*2 (11)*	*17 (24)*	*23 (28)*	*0.14*
*HIV -*	*23 (24)*	*16 (19)*	*29 (37)*	*0.069*
Sex with multiple partners	4 (2.6)	6 (2.9)	3 (1.5)	0.61
Experienced coercion	34 (22)	52 (25)	50 (25)	0.77
Experienced force	7 (4.5)	5 (2.4)	4 (2.0)	0.33
Sex for money or material benefits	2 (1.3)	8 (3.8)	2 (1.0)	0.096
Commercial sex work	1 (0.6)	0 (0)	1 (0.5)	0.54
About steady sexual partner (n = 538)				
Knows that partner has concurrent partners †	21 (14)	52 (26)	61 (32)	0.001
Circumcised partner^c^	136 (95)	169 (87)	161 (87)	0.030
*HIV +*	*17 (90)*	*55 (82)*	*60 (80)*	*0.38*
*HIV -*	*86 (98)*	*71 (93)*	*68 (91)*	*0.054*
Knows partner’s HIV-status	47 (33)	53 (27)	74 (39)	0.035
*HIV +*	*5 (26)*	*19 (27)*	*43 (55)*	*0.001*
*HIV -*	*32 (36)*	*26 (34)*	*27 (36)*	*0.94*

Data are provided as numbers (%) and median (range)

Some significant variables are further analysed and stratified for HIV infection

^a^Older women reported significantly less often to have concurrent sexual partners than women of 25–34 years (*p* < 0.001) and 18–24 years (*p* < 0.05)

^b^Partner >10y older was reported more often by older women (>34 years) compared to younger women (*p* < 0.05)

^c^Young women (<25 years) reported more often a circumcised partner than older women (*p* < 0.05)

*The association of women visiting bars of age <25 vs. 25–34 years and <25 vs. >34 years were significant, however no significant association was observed for women 25–34 vs. >34 years

^The association of women practicing fellatio of age <25 vs. >34 years and 25–34 vs. >34 years and were significant, however no significant association was observed for women <25 vs. 25–34 years

†The association of women knowing their partner has concurrent partners of age <25 vs. 25–34 years and <25 vs. >34 years were significant, however no significant association was observed for women 25–34 vs. >34 years

Condom use during last sex act was low among all age groups and not significantly different. (37 % for 18–24, 35 % for 25–34 and 37 % for >34; *p* = 0.86) However, when asked for reasons for not using a condom, women in the older age group (35–49 years) were more likely to report that the partner did not want to use them (51 %) than women in the younger age groups (37 % for 25–34 years old, 29 % for 18–24 years old; *p* < 0.001 for both comparisons). This association was only manifested for HIV-uninfected women (*p* < 0.01) whereas there was no association for HIV-infected women (*p* = 0.97). Women in the younger age group (<25 years) reported that their partner did not want to use condoms (29 %), that they trusted their partner (19 %), pregnancy wish (11 %) and non-availability (12 %) as main reasons not to use a condom.

### Ethnic origin and sexual behaviour

Sotho women were more likely to report concurrent sexual relationships whereas intravaginal cleansing and vaginal scarring practices were more often reported by Shangaan women (Table 3). About the steady partner, Shangaan women reported more often that they knew the HIV status of their partner (29 % for Shangaan vs. 22 % for Sotho; *p* = 0.028), or that he was HIV-infected (16 % for Shangaan vs. 6.9 % for Sotho; *p* = 0.001) and a trend was observed for Shangaan women reporting more often that their partner had concurrent sexual partners (29 % for Shangaan vs. 22 % for Sotho; *p* = 0.07). Sotho women reported more often having a circumcised partner (93 % for Sotho vs. 85 % for Shangaan; *p* = 0.007).

**Table 3:** Characteristics of sexual behaviour of Sotho and Shangaan women (*n* = 570)

Characteristic	Sotho women (*n* = 310)	Shangaan women (*n* = 260)	*p*-value
General characteristics			
Age (years)	29 (18–49)	32 (18–49)	0.19
HIV infected	90 (40)	88 (42)	0.63
Hormonal contraceptives	124 (40)	100 (39)	0.74
Intravaginal cleansing	49 (16)	86 (33)	<0.001
Vaginal scarring practice	10 (3.2)	25 (9.7)	0.001
Sexual practice in past 6 months			
Currently has a steady partner	293 (95)	245 (95)	0.91
Currently has occasional partner(s)	40 (13)	23 (8.9)	0.13
Days since last sex act	8 (0–180)	9 (0–180)	0.42
Condom use during last sex act	116 (38)	90 (35)	0.49
Practices fellatio	45 (15)	31 (13)	0.44
Receptive anal intercourse	16 (5.4)	10 (4.2)	0.52
Sexual contact in past 12 months			
Concurrent sexual partners	60 (19)	27 (11)	0.003
Sexual partner > 10 years older	67 (22)	59 (23)	0.68
Sex with multiple partners	7 (2.3)	6 (2.3)	0.97
Sex for money or material benefits	9 (2.9)	3 (1.2)	0.15
Commercial sex work	1 (0.3)	1 (0.4)	1.0

Data are provided as numbers (%) and median (range). HIV, Human Immunodeficiency Virus

### HIV-status and sexual behaviour

HIV-status was reported as positive by 178 (31 %) women and negative by 257 (45 %). One hundred and thirty-five women (24 %) were classified as unknown HIV-status and were excluded from this particular sub-analysis. HIV-infected women were older than those without HIV infection, reported a higher number of lifetime sexual partners, and were more likely to report condom use during last sex act and to have a known HIV-infected steady partner (Table 4). Hormonal contraceptives use (28 % for HIV-infected vs. 45 % for HIV-uninfected; *p* < 0.001), fellatio and a circumcised sexual partner were reported less often by HIV-infected women.

**Table 4:** Characteristics of sexual behaviour for HIV-infected and HIV-uninfected women (*n* = 435)

Characteristic	HIV-infected (*n* = 178)	HIV-uninfected (*n* = 257)	*p*-value
General characteristics			
Ethnicity (Sotho vs. Shangaan)	90 (51)/88 (49)	136 (53)/121 (47)	0.63
Age	34 (20–49)	28 (18–49)	<0.001
Hormonal contraceptives	50 (28)	116 (45)	<0.001
Intravaginal cleansing	42 (24)	73 (28)	0.29
Visits bars	20 (11)	36 (14)	0.41
Alcohol use [before sex intercourse]	15 (8.6)	26 (10)	0.58
Lifetime number of sexual partners	4 (1–30)	3 (1–15)	<0.001
Sexual practice in past 6 months			
Currently has a steady partner	167 (95)	243 (95)	0.99
Currently has occasional partner(s)	24 (14)	25 (9.8)	0.21
Condom use during last sex act	91 (51)	68 (27)	<0.001
Practices fellatio	16 (9.5)	46 (19)	0.010
Receptive anal intercourse	6 (3.6)	15 (6.1)	0.26
Sexual contact in past 12 months			
Concurrent sexual partners	33 (19)	33 (13)	0.095
Sexual partner > 10 years older	42 (24)	68 (27)	0.59
Sex with multiple partners	3 (1.7)	6 (2.3)	0.74
Experienced coercion	42 (24)	76 (30)	0.18
Experienced force	4 (2.3)	7 (2.7)	1.0
Sex for money or material benefits	4 (2.3)	7 (2.7)	1.0
Commercial sex work	1 (0.6)	1 (0.4)	1.0
About steady sexual partner (n = 538)			
Knows that partner has concurrent partners	46 (28)	55 (23)	0.26
Circumcised partner	132 (82)	225 (94)	<0.001
Knows partner’s HIV-status	67 (40)	85 (35)	0.30
Known HIV-infected partner	55 (33)	2 (0.8)	<0.001

Data are provided as numbers (%) and median (range). HIV, Human Immunodeficiency Virus

### Condom use

Two hundred and six women (36 %) reported male and or female condom use during last sex act: 201 used a male, 2 a female and 3 women used both condoms. The top three reasons given for why a condom was not consistently used were: ‘partner does not want to’ by 184 (39 %), ‘trust partner’ by 73 (16 %) and ‘pregnancy wish’ by 58 (13 %).

Pregnant women and women who reported engaging in vaginal cleansing and RAI were less likely to report condom use during last sex act whereas single, employed women and those with occasional sexual partners were more likely to do so (Table 5). The association of condom use with pregnancy was manifested for HIV infected women (30 % for pregnant vs. 56 % for non-pregnant women; *p* = 0.011) while a trend was observed for HIV uninfected women (16 % for pregnant vs. 29 % for non-pregnant women; *p* = 0.057) and manifested for women who did not report concurrent sexual partners (20 % for pregnant vs. 38 % for non-pregnant women; *p* = 0.002) whereas there was no association observed for women with concurrent sexual partners (35 % for pregnant vs. 45 % for non-pregnant women; *p* = 0.47).

**Table 5:** Factors associated with condom use during last sex act

Characteristic	Condom use	No condom use	OR (95 % CI)	*p*-value
Age	30 (18–49)	30 (18–49)	–	0.66
Ethnicity				
Sotho	116 (38)	193 (63)	–	0.49
Shangaan	90 (35)	169 (65)		
HIV-status				
HIV-infected	91 (51)	86 (49)	2.9 (2.0–4.4)	<0.001
HIV-uninfected*	68 (27)	189 (74)		
Marital status				
Single	127 (42)	174 (58)	*	0.001
Married/engaged	66 (28)	173 (72)		
Divorced/widow	12 (44)	15 (56)		
Employed				
Yes	62 (43)	81 (57)	1.5 (1.0–2.2)	0.040
No	143 (34)	280 (66)		
Pregnant				
Yes	22 (22)	76 (78)	0.46 (0.28–0.76)	0.002
No	180 (39)	285 (61)		
Hormonal contraceptives				
Yes	76 (34)	147 (66)	–	0.41
No	129 (38)	215 (63)		
Intravaginal cleansing				
Yes	36 (27)	99 (73)	0.57 (0.37–0.87)	0.009
No	168 (39)	263 (61)		
Alcohol use [before sex intercourse]				
Yes	18 (32)	38 (68)	–	0.51
No	186 (37)	322 (63)		
Lifetime number of sexual partners	3 (1–15)	3 (1–30)	–	0.16
Concurrent sexual partners				
Yes	38 (44)	49 (56)	–	0.12
No	168 (35)	312 (65)		
Currently has a steady partner				
Yes	193 (36)	344 (64)	–	0.61
No	11 (41)	16 (59)		
Occasional partner(s)				
Yes	29 (46)	34 (54)	–	0.086
No	175 (35)	325 (65)		
Receptive anal intercourse				
Yes	3 (12)	23 (89)	0.22 (0.064–0.73)	0.007
No	192 (38)	318 (62)		
Fellatio				
Yes	29 (38)	47 (62)	–	0.72
No	166 (36)	295 (64)		

Data are provided as numbers (%) and median (range). OR, odds ratio; CI, confidence interval; HIV, Human Immunodeficiency Virus

*p < 0.001 for single vs. married (OR 1.9; 95 % CI 1.3–2.8); *p* = 0.82 for single vs. divorced and *p* = 0.07 for married vs. divorced

Also, women who had experienced coercion and those who had sex with a partner more than 10 years older were less likely to use a condom. (*p* <0.001 and *p* = 0.014, respectively).

## Discussion

This study is one of the few providing insight into sexual behaviour of women in rural South Africa and the first to assess women in a region with predominantly people of Sotho and Shangaan ethnic origin. We show differences in sexual behaviour for different ages, ethnicity and by HIV-status. These differences together with the associations observed with condom use illustrate the complexity of sexual behaviour in this region. Also, they provide an entry point for further discussion towards the design of tailor-made prevention strategies for these different groups of women. The sample of women included in this evaluation is considered representative for ethnic composition, age and HIV status of women visiting PHC facilities in this area.

The relation between age and sexual behaviour is well documented and confirmed in this study. Younger women are known to engage in higher risk sexual behaviour which is reflected in our study by the higher number of concurrent sexual partners. In addition, potential lifestyle differences (e.g. more bar visits) were observed.

Recently, a study from Kenya showed considerable ethnic disparities in HIV prevalence arising from sexual behaviour [13]. The relevance of race (black, white, and coloured) as a determinant of sexual behaviour has been reported before, but information about ethnic groups in South Africa is limited [17, 18, 21]. A recent study presented characteristics of sexual behaviour by isiXhosa women but no data are available for women of Sotho and Shangaan origin [16, 17, 24, 25]. Sotho women were more likely to report concurrent sexual partners whereas intravaginal cleansing, vaginal scarring and knowledge of HIV-status of the steady partner were more frequent among Shangaan women. This may reflect a bias in recruitment although there was no specific selection and participants were representative of those visiting a primary healthcare facility on a random day. On the other hand, these differences may be an important observation to warrant further research which could, hypothetically, result in a design of prevention efforts to be implemented at the same facilities where our study population was recruited: For example, Sotho women could be targeted to promote safe sex in (concurrent) relationships whereas promoting safe sex to the woman’s steady sexual partner and safe vaginal practices should perhaps feature more dominantly in areas where the predominant ethnic group is Shangaan. Overall, knowledge of the partner’s HIV-status was low among women of both ethnic groups. Discussing safe sex and sexual practice may be taboo within relationships in this region and understanding the influence of cultural practices, social beliefs, financial dependency and local media is required to inform the design of intervention strategies [26–29].

The association of HIV-infected status and sexual behaviour is reasonably well-documented, but information from rural Africa and rural South Africa is limited. Our study shows that HIV-infected women reported a higher lifetime number of sexual partners, were more likely to use a condom during last sexual act, and to have a known HIV-infected partner whereas HIV negative women were more likely to use hormonal contraceptives and practice fellatio. Having a circumcised partner was significantly more frequently reported by HIV-negative women. Most of these associations have been reported before [3, 12, 19, 30]. The lower frequency of practising fellatio by HIV-infected women has been reported once before [20]. This could relate to a difference in age as HIV-infected women were older and young women were more likely to engage in fellatio or a change, e.g. reduction, in sexual activity following a positive HIV-test. The findings in our study suggest an essential difference in sexual behaviour related to HIV-status, but the study design did not allow determining temporality of this relationship.

We determined factors associated with (non-)use of condoms to support the development of prevention tools [31]. Women reporting condom use during the last sex act were more likely to be single, employed, HIV-infected and have occasional sexual partners while non-users were more likely to be pregnant, engage in RAI and intravaginal cleansing, experience coercion and have a partner more than 10 years older. These factors are well known in African populations where condom use within the marriage is unusual and pregnancy frequently associated with lower uptake of condom use [19, 32]. While alcohol use before intercourse is a known factor of sexual risk behaviour, there was no difference between condom uptake between women using alcohol before intercourse or not [33, 34]. Men in age-disparate relationships, i.e. young woman with substantially older male partner, are known to use their age-related power to undermine young women’s preferences for safer sex [35]. Efforts aimed at reducing age mixing are complicated, but may decrease the risk of HIV and STIs among young women in a traditional setting such as ours [36]. In summary it appears that non-use of condoms is related to a more traditional lifestyle and such areas may be the first target of safe sex promotion and HIV/STI prevention campaigns.

This study has several limitations. First, this study design does not allow for assessment of temporality of the observed associations and the results should be interpreted with caution. Sexual behaviour is a complex entity and we can only provide some degree of insight. Most importantly, the groups compared in this analysis could provide insight into different target groups and entry points of prevention efforts. Second, we used self-reported age, HIV-status and ethnic group. We tried to limit the effect of self-report by using strict criteria for classification of HIV-status. Third, reporting and desirability bias may have occurred although we tried to create as safe as possible environment. This could play a role in particular when considering the positive association of condom use with HIV-positive status: women are reminded regularly when collecting antiretroviral therapy to use condoms. Selection bias may have occurred and extrapolation of information should be done cautiously as we only included women visiting PHC facilities. These women are of low socio-economic status, demonstrated by the high unemployment rate reported in our study compared to the average in this area, and cannot afford private healthcare. However, the study was conducted at a representative sample of PHC facilities in the district, which provide prominent locations to implement preventions campaigns.

## Conclusions

More information about heterosexual sexual behaviour in Africa is warranted to determine the relevance and potential impact of efforts aimed at modifying sexual behaviour for HIV and STI prevention. In this regard, prevention efforts at a local level should take the population’s composition of age, ethnicity and HIV-status into account when designing a tailor-made package that combines biomedical approaches with behavioural modification efforts.

## Abbreviations

ART, antiretroviral therapy; HIV, Human Immunodeficiency Virus; PHC, primary healthcare; RAI, receptive anal intercourse; STI, sexually transmitted infection
